# Collateral Presentation of Malaria and Dengue Viral Hemorrhagic Fever: A Rare Case

**DOI:** 10.7759/cureus.4050

**Published:** 2019-02-11

**Authors:** Qazi Arsalan, Laila Tul Qadar, Rohan Kumar Ochani, Faryal Tahir, Zainab Majid

**Affiliations:** 1 Internal Medicine, Dow University of Health Sciences, Karachi, PAK

**Keywords:** dengue, co-infection, concurrent infection, malaria, mosquito borne diseases

## Abstract

Cases on concurrent infection of dengue and malaria are uncommon in Pakistan. Dengue and malaria are the two very common, mosquito-borne infections, which may have significant morbidity and mortality if not managed properly. Concurrent infections of dengue and malaria are rare due to the different habitats of its vectors and activities of different bearer mosquitoes. The first case was reported in 2005. Since then, several co-existing infections have been reported consisting of the dengue virus (DENV) and the malaria protozoans. Symptoms of each infection may be disguised by a coincident second infection, resulting in delayed treatment and severe complications. We report a case of concurrent malaria and dengue viral hemorrhagic fever in a 19-year-old male.

## Introduction

Several tropical vector-borne infections continue to cause a rising incidence of morbidity and mortality in resource-restricted nations. Two of those infections are: a parasitic disease malaria and a viral disease dengue. Malaria is caused by a female mosquito, Anopheles; while dengue is caused by Aedes aegypti mosquito. In the past, the presence of a co-infection in an individual is sporadically reported [1, 2]. Both of the diseases cause an acute pyrexial illness; however, only malaria can cause a chronic fever. Here, we present a case of a young male with overlapping symptoms and the challenging enigma of diagnosis to aware physicians in the endemic areas of Pakistan for the possibility of malaria and dengue co-infection.

## Case presentation

A 19-year-old male, a resident of Mirpur with no known co-morbidities, presented to the emergency department (ED) of Dr. Ruth KM Pfau, Civil Hospital Karachi (CHK) in September 2018 with a history of fever, dizziness, generalized weakness and bleeding of gums since the past three days. According to the patient, he suddenly developed a continuous fever of 102°F, which was sporadic in nature and associated with chills and rigors. The fever temporarily alleviated with intake of antipyretics. He also experienced occasional bleeding from gums and dizziness with generalized weakness, for which he sought symptomatic treatment from a local health care facility, but the symptoms worsened. Therefore, he was then referred to CHK. No history of bleeding from any other site, nor hematemesis or black tarry stools was present. The patient revealed a decreased appetite and past addiction to tobacco.

On examination (O/E), the patient was of average height and built, comfortably lying on the bed, and well oriented to time, place and person. Initial vitals included blood pressure (BP) 120/70 mmHg, a regular pulse of 90 beats/min and a respiratory rate of 20 breaths/min. The patient was anemic and dehydrated. He had a soft, non-tender, non-distended abdomen without hepatosplenomegaly, and bowel sounds were audible with a rate of 3-4/min. All other systems were unremarkable.

Blood investigations revealed a hemoglobin (Hb) of 4.5 gm/dl, mean corpuscular volume (MCV) of 108.5 fl, mean corpuscular hemoglobin concentration (MCHC) of 35.4 gm/dl, total leukocyte count (TLC) of 2.7 x 109 L, hematocrit (HCT) of 12.7%, platelet count (PLT) of 12 x 109/L. The clotting profile showed an international normalized ratio (INR) of 1.11, while prothrombin time (PT) and activated partial thromboplastin time (aPTT) were 11.1 and 21.7 seconds, respectively. The various lab investigations conducted, including those for hepatitis B surface antigen and hepatitis C antibody, both came out normal, as did his X-ray chest, renal and liver function tests, spot urine examination and ultrasound of the abdomen. The need for conducting specific investigations like hepatitis and chest X-ray were to exclude any other likely cause of the fever and presence of an infection. The electrolytes were within the normal range as well. Upon serology testing, dengue antigen came out to be reactive while that of dengue virus-specific antibodies, immunoglobulin M (IgM) and immunoglobulin G (IgG), were not reactive. Malarial parasite (MP) and MP immunochromatographic test (ICT) came out to be positive for Plasmodium (P) vivax with 7% reticulocytes (RET). Therefore, a diagnosis of concurrent malaria and dengue infection was established.

The patient was treated with an oral combination of artemether and lumefantrine 80/480 twice daily for three days, along with oral acetaminophen two tablets if needed. Additionally, he was intravenously (IV) given tranexamic acid 5 mg when necessary and 1000 ml sodium chloride at the rate of 80 ml/hour. Two units of packed cells and six units of platelets were transfused alongside. Malaria and dengue were treated as separate entities with the above-mentioned treatment.

## Discussion

Dengue and malaria are the most prevalent arthropod-borne diseases with an estimated global incidence of 390 million and 214 million cases a year, respectively [3].

Although usually their co-infection is masked as a mono infection; however, in 2005, Charrel et al. were successful in publishing the first dengue and malarial coherent infection [4]. Once the disease is diagnosed for one infection, the other must not be ruled out until it has been screened. This is shown by a study conducted in Cayenne Hospital, French Guiana, where out of 1,723 consecutive febrile patients, a total of 238 patients had dengue, 393 had malaria, and 17 had both [5]. The comparison between our case and a typical concurrent malaria and dengue infection is shown in Table 1.

**Table 1:** Comparing the presentation of a typical malaria and dengue concurrent infection in our case.

CHARACTERISTICS	TYPICAL PRESENTATION	PRESENTATION OF CASE
Mode of transmission	Mosquito borne (Anopheles + Aedes)	Mosquito borne (Anopheles + Aedes)
Fever	Acute febrile illness	Acute febrile illness
Myalgia	Common	Positive
Shock	Possible	Negative
Blood parasite	Positive	Positive
Atypical lymphocytosis	Usually positive	Positive
Hemoconcentration	Usually positive	Negative
Thrombocytopenia	Usually positive	Positive
Bleeding	Possible	Positive
Hemolysis	Rare	Negative
Treatment	Antimalarial drug + fluid therapy	Antimalarial drug + fluid therapy

A recent study showed that, in the last 15 years, the most number of malaria and dengue co-infection cases were reported from Pakistan. This is typical because the exponential rise in the cases is due to the rapidly changing environment, leading to an increased transmission period for both dengue and malaria. The most common species present in cases of malaria co-infections are P. falciparum and P. vivax, whereas, for dengue co-infections, dengue serotype 4 is the most common horrifying serotype [6].

A case series study conducted in Jinnah Postgraduate Medical Centre, Karachi from September 2007 to January 2008 showed that the diagnosis of dual infections could be made on the basis of history and clinical examination with the help of hematological results. This study included a total of 114 patients, out of which, 23.21% had dual dengue and malaria co-infection (dengue IgM and MP positive), whereas 30.35% patients were negative for both the markers, but were strongly suspected for dengue hemorrhagic fever (DHF) and malaria on the basis of hematology and clinical results.

Therefore, it is recommended that all patients suspected for dual infections should be treated collaterally for dengue and malaria in the endemic areas [7]. According to a case reported in Srinagar, India, a patient who had a simultaneous co-infection of dengue and malaria, positive for P. vivax and P. falciparum, was orally given 600 mg of chloroquine phosphate, reduced to 300 mg after six hours and then 300 mg daily for another two days. The response to treatment was good as the fever mitigated and the general condition of the patient improved quickly. Additionally, he was also put on a 30 mg oral dose of primaquine daily for 14 days as a profound cure after achieving normal levels of glucose-6-phosphate dehydrogenase (G6PD) [8].

A cross-sectional study conducted in Brazil showed the prevalence of malaria and dengue co-infection in a large sample taken. Out of the 1578 patients admitted to the hospital in a span of three years, 176 patients and 584 patients had P. vivax malaria and dengue fever mono-infection, respectively; while 2.8% (n = 44) patients were co-infected with both malaria and dengue. This study also explained the basis of diagnosing dengue virus, concluding the importance of diagnosing such infections precisely. Firstly, the IgM antibodies were detected; secondly, non-structural (NS1) protein was detected using Platelia Dengue NS1 antigen kit and finally, reverse transcriptase-polymerase chain reaction was used to identify the viral serotype [9].

General practitioners (GPs) in Pakistan lack the necessary knowledge regarding prevalent concurrent infectious diseases and their treatments, and misdiagnose the cases as separate malaria or dengue attack. The severity of co-infections is much more than single infections as the patients have a higher risk of being anemic and thrombocytopenic [10]. Therefore, there is a dire need to inform GPs regarding the management of such cases [11].

## Conclusions

The need for reporting this case is to highlight the presence of co-existing infections of malaria and dengue affecting the health of many people in endemic areas of Pakistan. Lack of knowledge regarding specific investigations and management may lead to catastrophic consequences. Thus, modern monitoring vector dynamics will be useful to determine the presence of such vectors and to formulate strategies with a huge emphasis on inhibition of vectors. This may lead to complete eradication in the future and consequently, reduce the transmission of infections. Prompt actions should be taken for educating the concerned masses about how to manage and treat these co-infections efficiently.

## References

[1] Shah PD, Mehta TK (2017). Evaluation of concurrent malaria and dengue infections among febrile patients. Indian J Med Microbiol.

[2] Wiwanitkit V (2011). Concurrent malaria and dengue infection: a brief summary and comment. Asian Pac J Trop Biomed.

[3] Chong SE, Mohamad Zaini RH, Suraiya S, Lee KT, Lim JA (2017). The dangers of accepting a single diagnosis: case report of concurrent Plasmodium knowlesi malaria and dengue infection. Malar J.

[4] Charrel RN, Brouqui P, Foucault C, de Lamballerie X (2005). Concurrent dengue and malaria. Emerg Infect Dis.

[5] Carme B, Matheus S, Donutil G, Raulin O, Nacher M, Morvan J (2009). Concurrent dengue and malaria in Cayenne Hospital, French Guiana. Emerg Infect Dis.

[6] Salam N, Mustafa S, Hafiz A, Chaudhary AA, Deeba F, Parveen S (2018). Global prevalence and distribution of coinfection of malaria, dengue and chikungunya: a systematic review. BMC Public Health.

[7] Abbasi A, Butt N, Sheikh QH, Bhutto AR, Munir SM, Ahmed SM (2009). Clinical features, diagnostic techniques and management of dual dengue and malaria infection. J Coll Physicians Surg Pak.

[8] Mushtaq MB, Qadri MI, Rashid A (2013). Concurrent infection with dengue and malaria: an unusual presentation. Case Rep Med.

[9] Magalhães BM, Siqueira AM, Alexandre MA (2014). P. vivax malaria and dengue fever co-infection: a cross-sectional study in the Brazilian Amazon. PLoS Negl Trop Dis.

[10] Epelboin L, Hanf M, Dussart P, Ouar-Epelboin S, Djossou F, Nacher M, Carme B (2012). Is dengue and malaria co-infection more severe than single infections? A retrospective matched-pair study in French Guiana. Malar J.

[11] Thaver AM, Sobani ZA, Qazi F, Khan M, Zafar A, Beg MA (2011). Assessing the need for training: general practitioners’ knowledge, attitude and practice concerning dengue and malaria in Karachi, Pakistan. Int Health.

